# Supplementary material for: Design, Synthesis, and Antitumor Activity of Olmutinib Derivatives Containing Acrylamide Moiety
Source: Molecules. 2021 May 20;26(10):3041. doi: 10.3390/molecules26103041 (PMC8160875; doi:10.3390/molecules26103041)
Supplement: Supplementary file 1 [file molecules-26-03041-s001.zip › molecules-1177035-SI.pdf]

*Supplementary Materials*

# Design, Synthesis and Antitumor Activity of Olmutinib Derivatives Containing Acrylamide Moiety

**Xiaohan Hu, Sheng Tang, Feiyi Yang, Pengwu Zheng, Shan Xu †, Qingshan Pan * and Wufu Zhu ***

School of Pharmacy, Jiangxi Science & Technology Normal University, Nanchang 330013, China; huxiaohan1717@163.com (X.H.); shengtang_1219@163.com (S.T.); 18380539689@163.com (F.Y.); Zhengpw@126.com (P.Z.); Shanxu9891@126.com (S.X.)

* Correspondence: qingshan_pan@hnu.edu.cn (Q.P.); zhuwufu-1122@163.com or zhuwf@jxstnu.edu.cn (W.Z.)

† Tel.: +86-791-8380-2393 (S.X.).

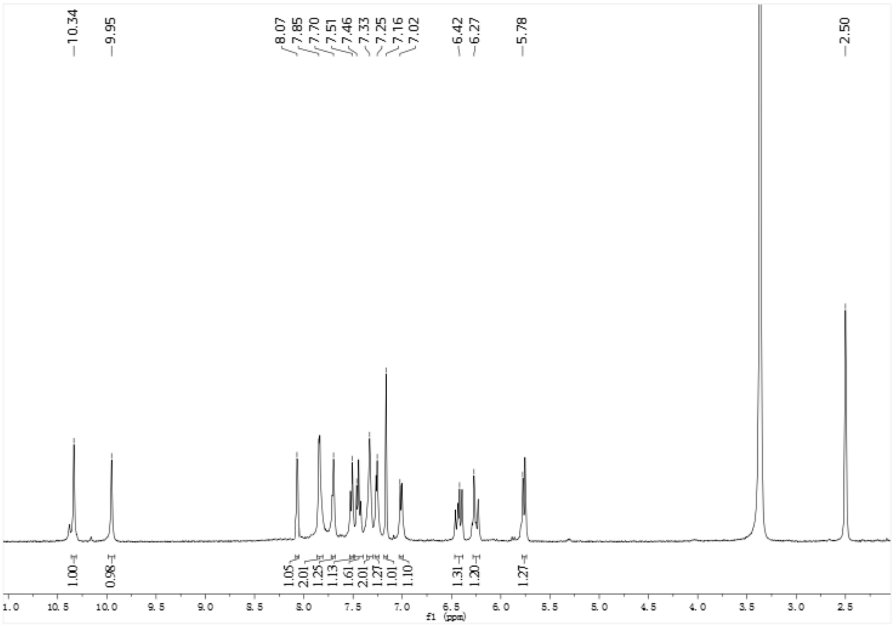

**Figure S1.** $^{1}$H-NMR spectrum of compound **H1.**

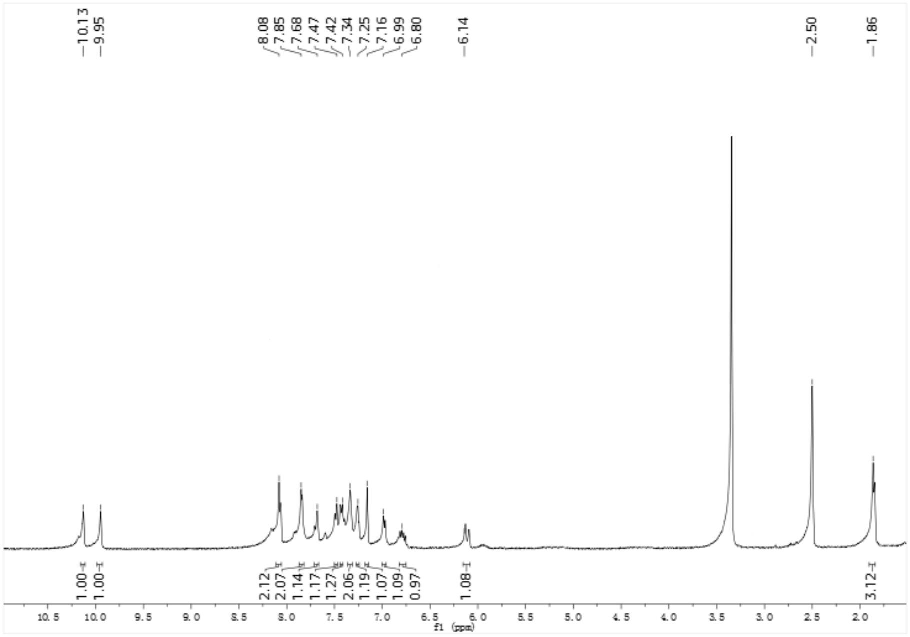

**Figure S2.** $^1$H-NMR spectrum of compound **H2.**

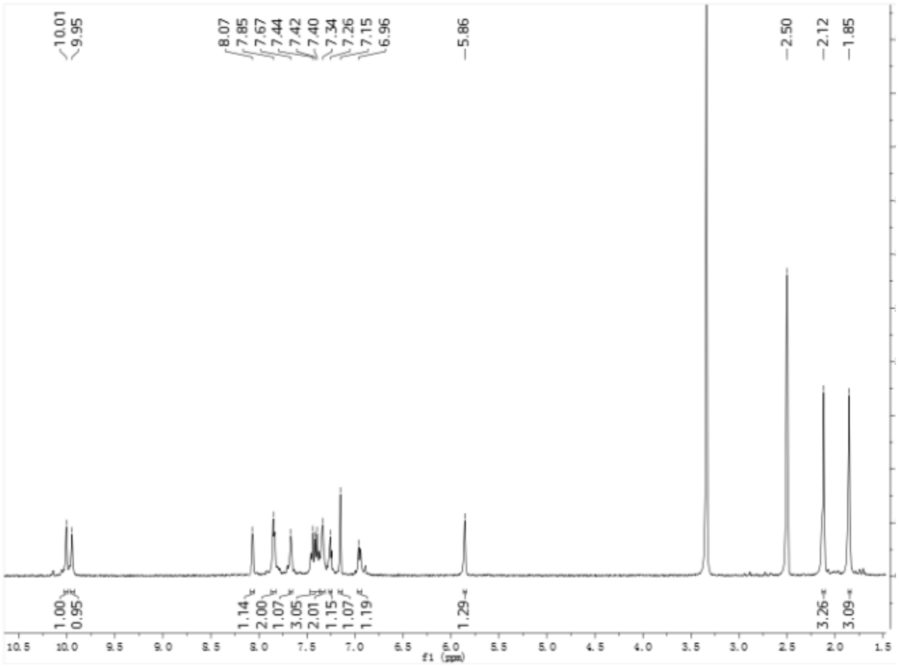


**Figure S3.** $^1$H-NMR spectrum of compound **H3.**

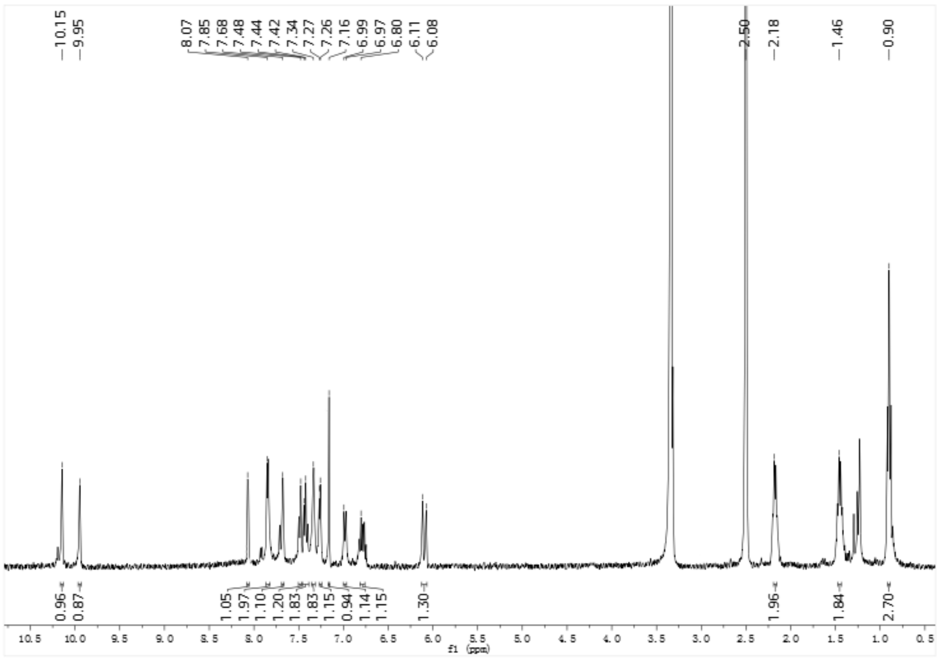


**Figure S4.** $^1$H-NMR spectrum of compound **H4.**

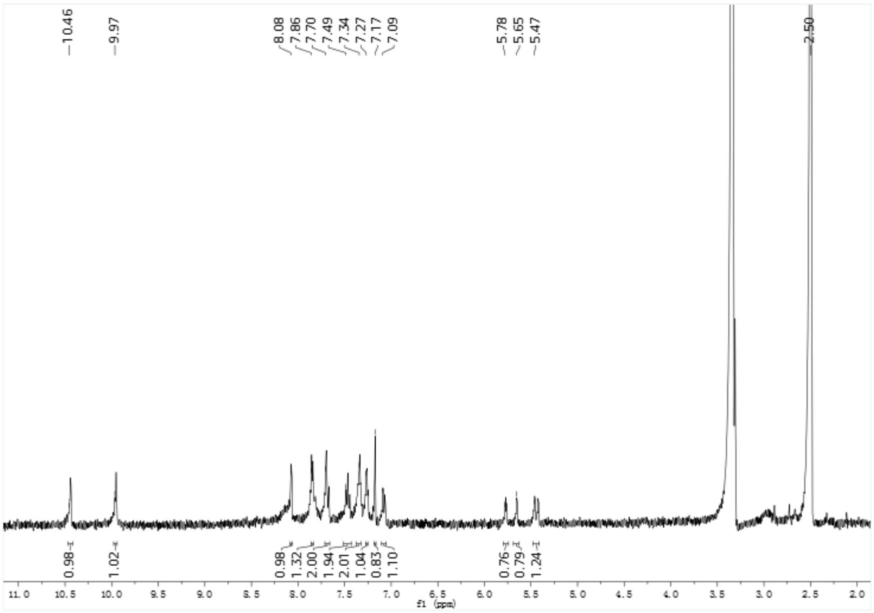


**Figure S5.** $^{1}H$-NMR spectrum of compound **H5.**

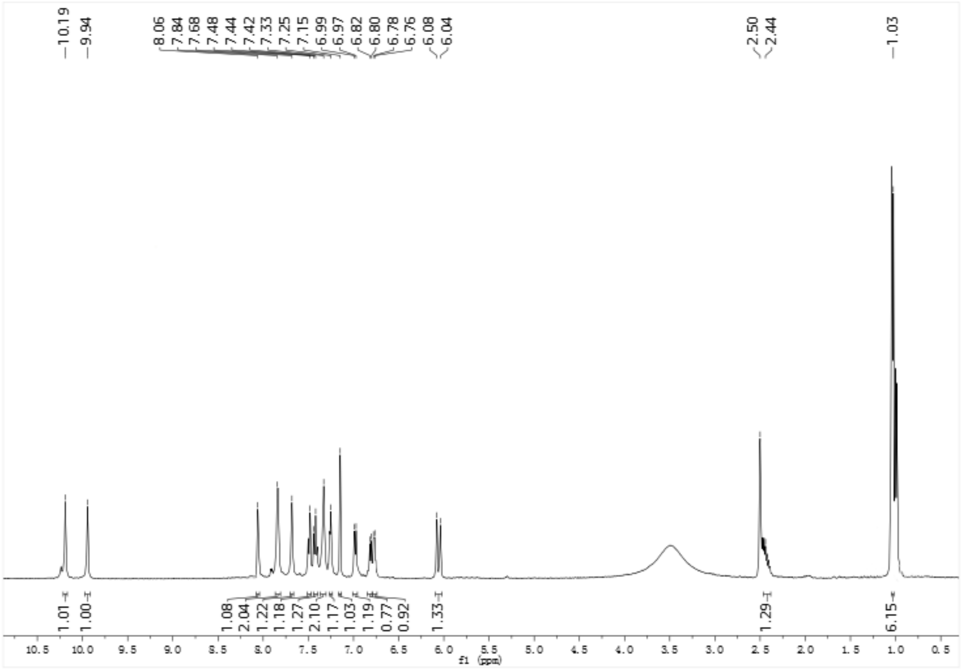


**Figure S6.** $^{1}H$-NMR spectrum of compound **H6.**

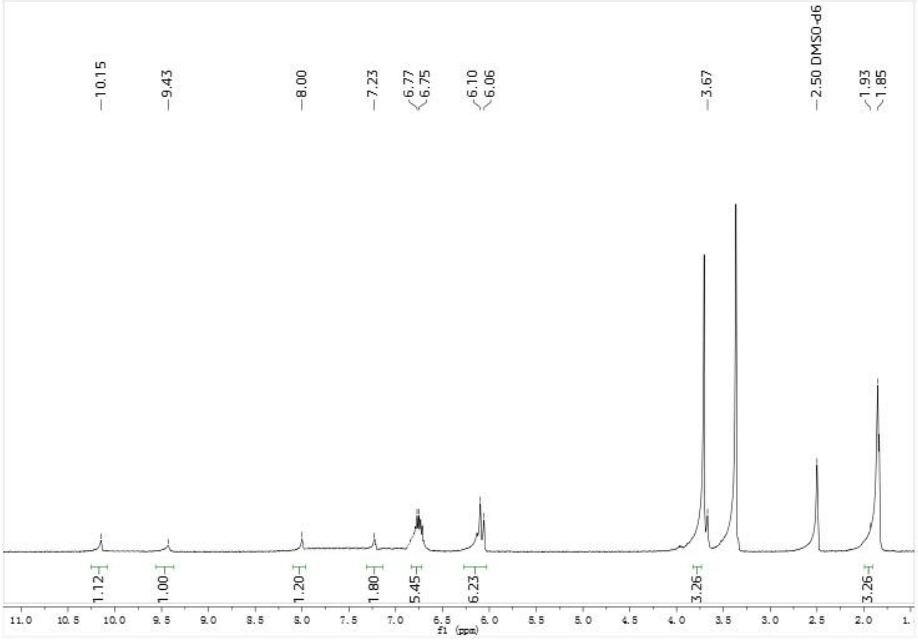


**Figure S7.** $^{1}$H-NMR spectrum of compound **H7.**

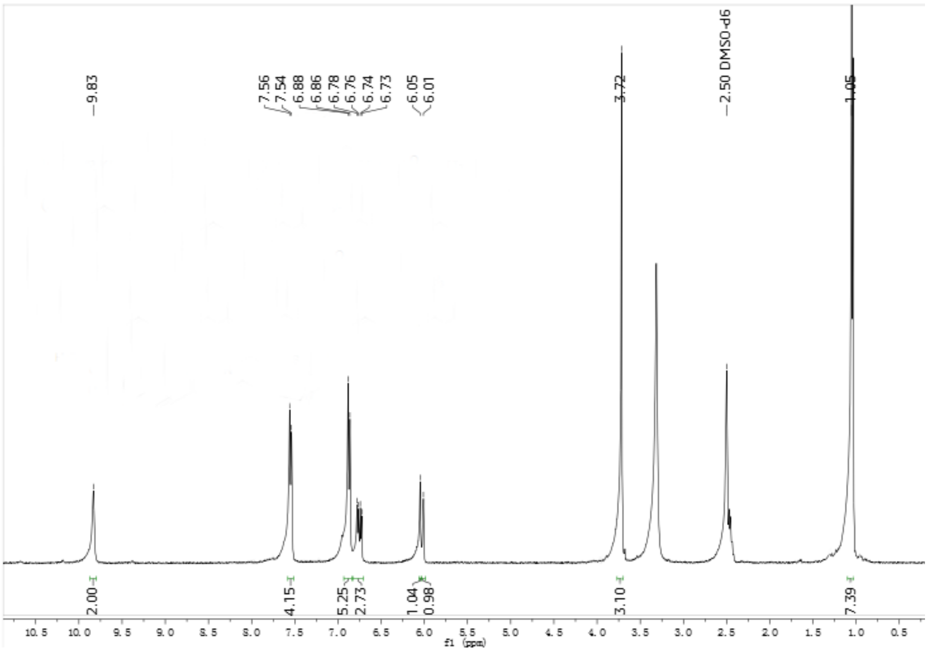


**Figure S8.** $^{1}$H-NMR spectrum of compound **H8.**

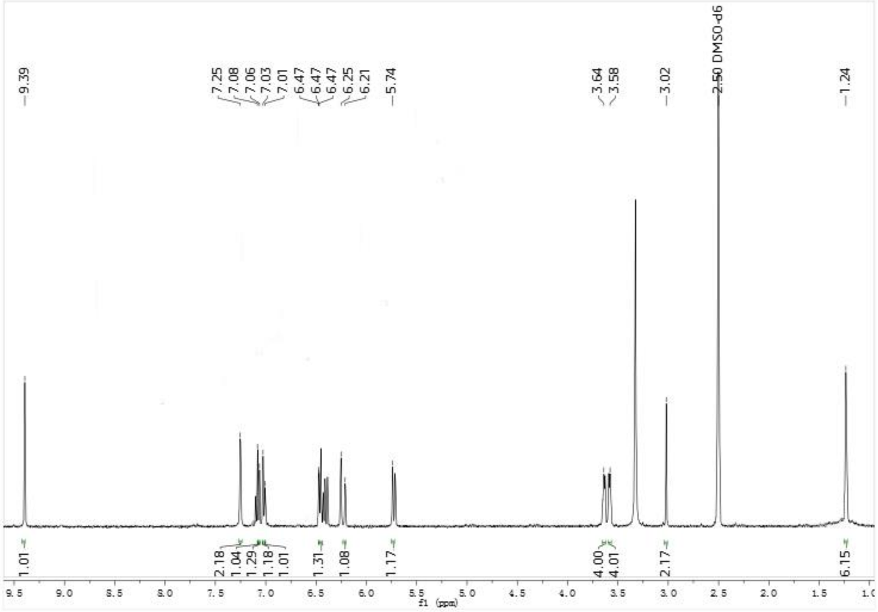


**Figure S9.** $^{1}$H-NMR spectrum of compound **H9.**

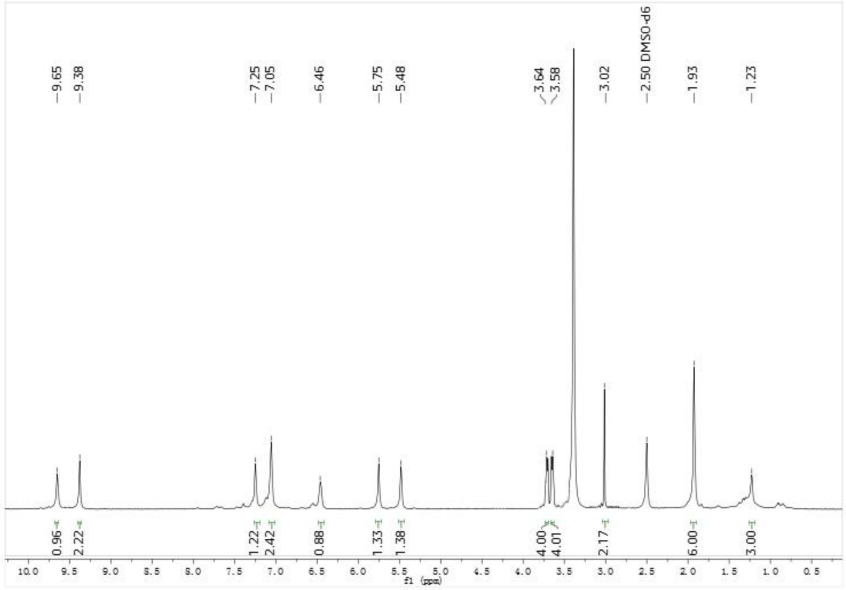


**Figure S10.** $^{1}$H-NMR spectrum of compound **H10.**

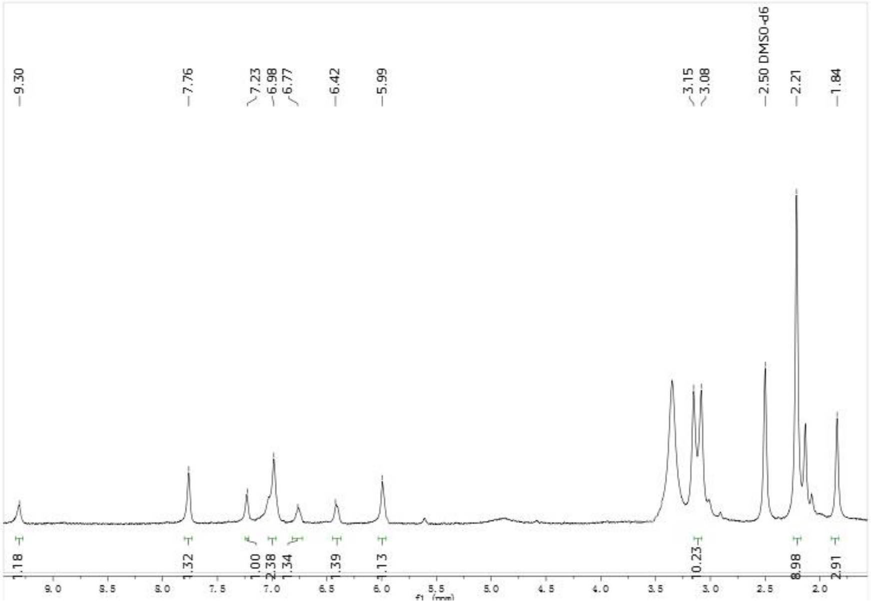


**Figure S11.** $^{1}$H-NMR spectrum of compound **H11.**

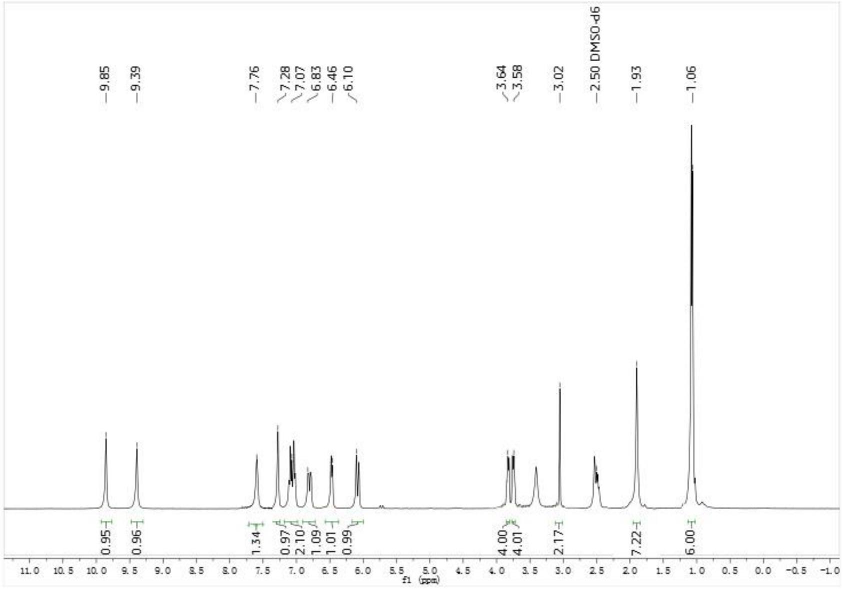


**Figure S12.** $^{1}$H-NMR spectrum of compound **H12.**

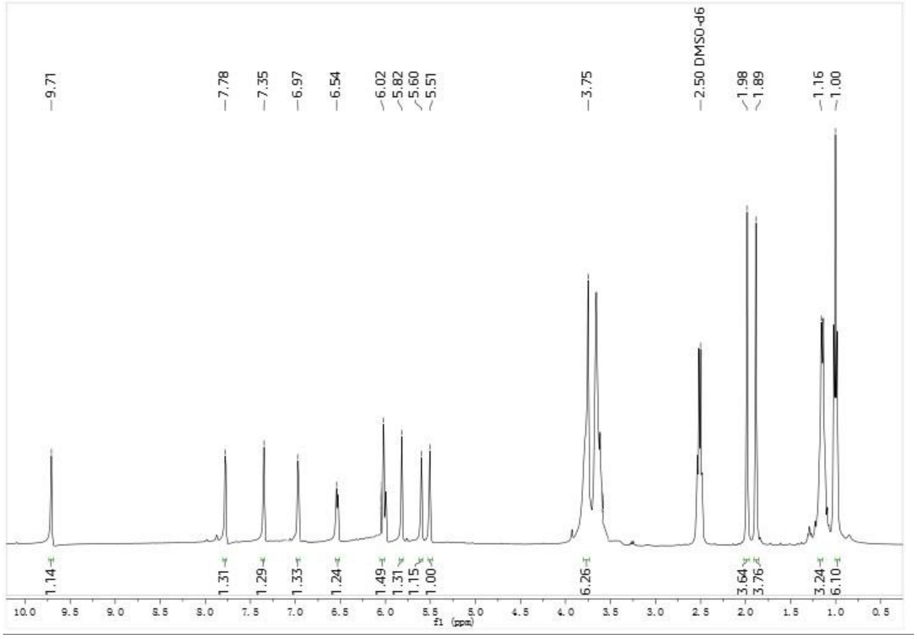


**Figure S13.** $^{1}$H-NMR spectrum of compound **H13.**

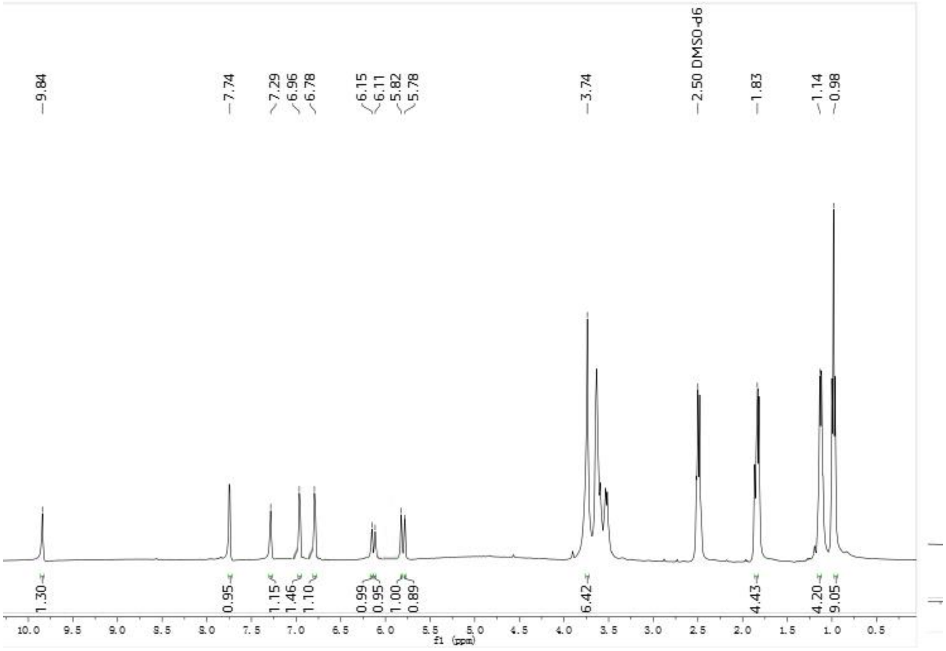


**Figure S14.** $^{1}$H-NMR spectrum of compound **H14.**

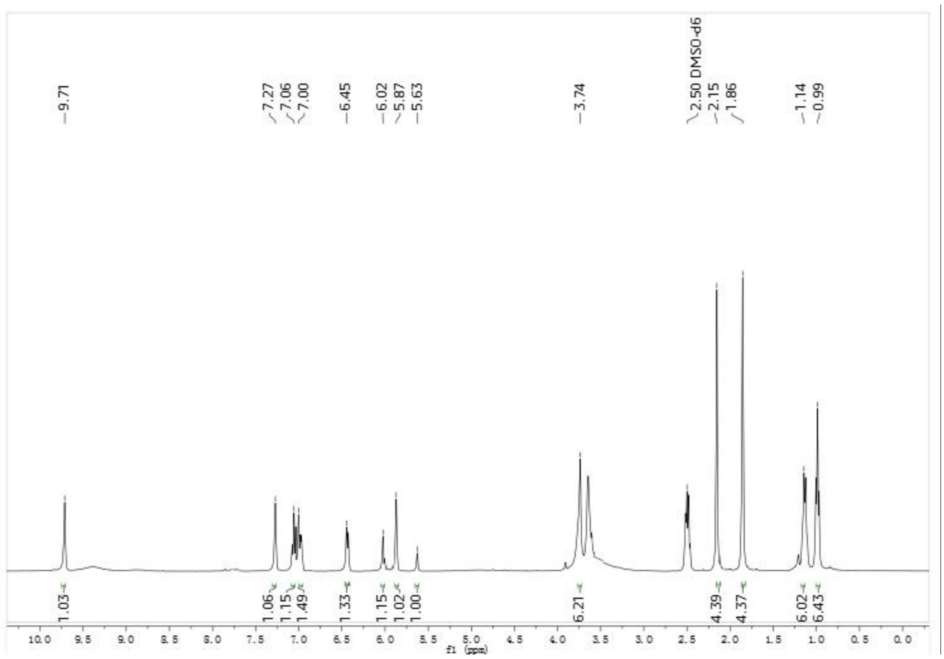

**Figure S15.** $^{1}$H-NMR spectrum of compound **H15.**

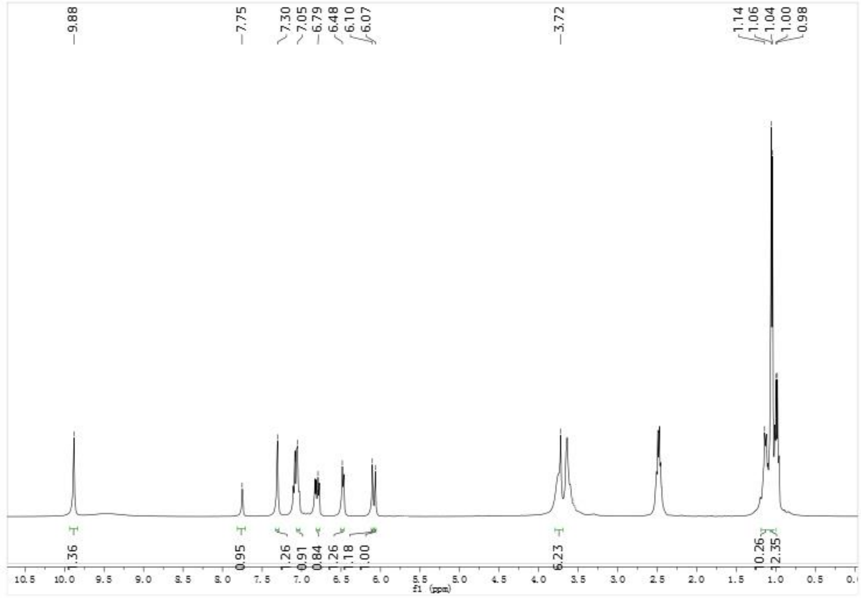

**Figure S16.** $^{1}$H-NMR spectrum of compound **H16.**

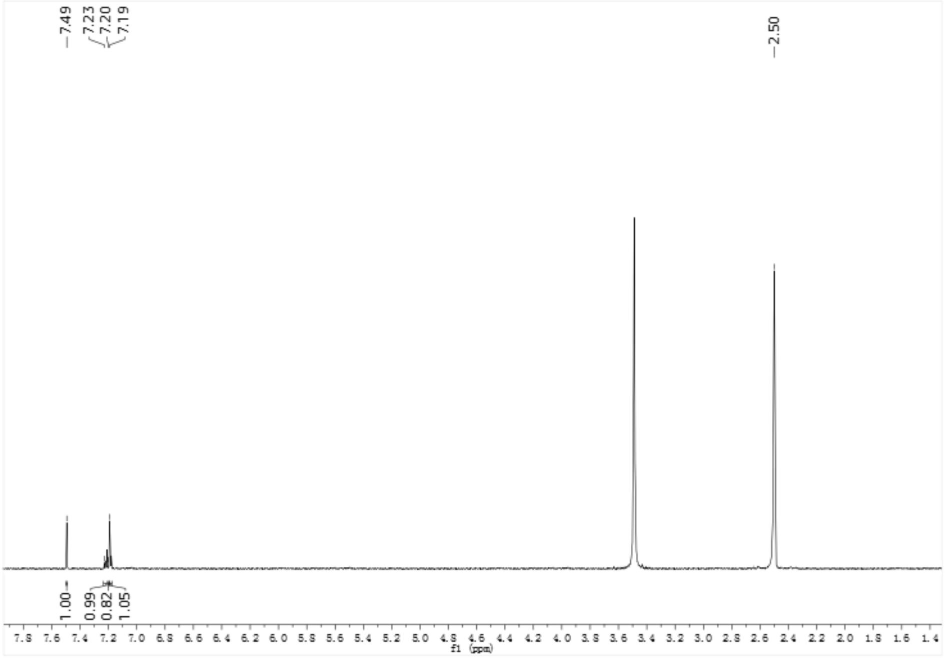


**Figure S17.** $^{1}$H-NMR spectrum of compound **8.**

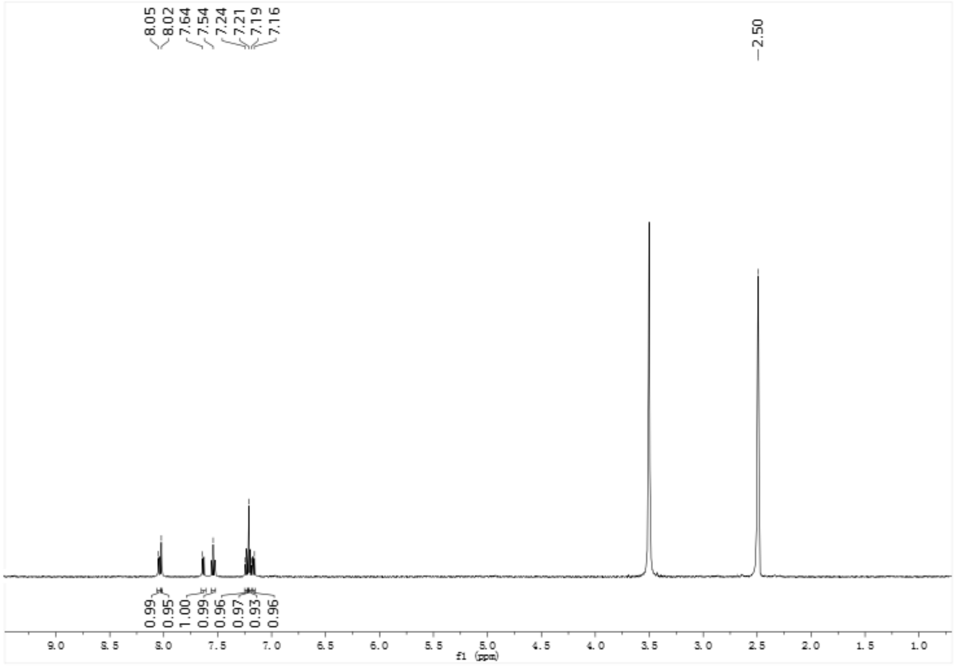


**Figure S18.** $^{1}$H-NMR spectrum of compound **9.**

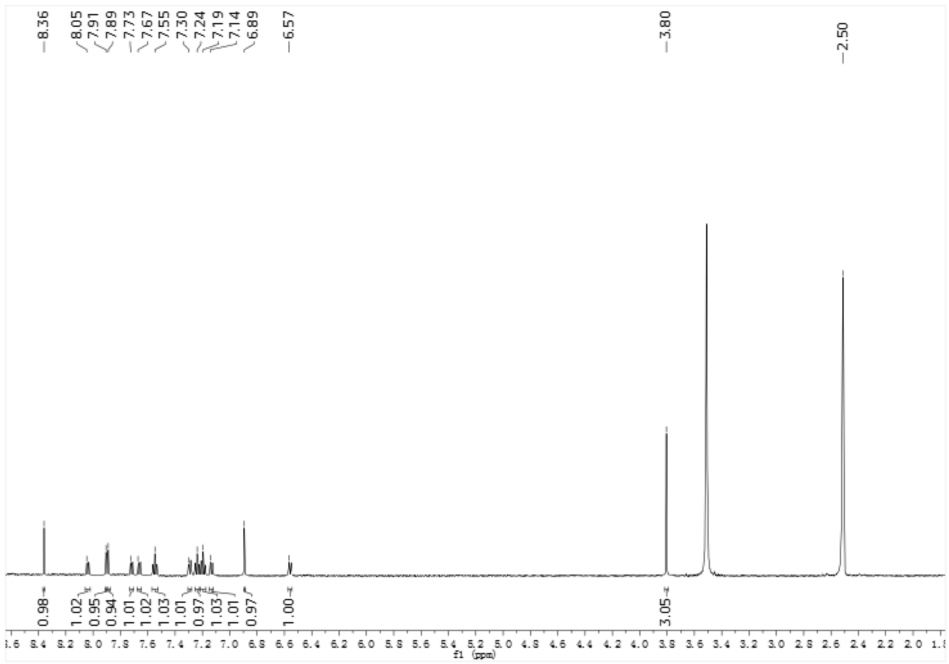


**Figure S19.** $^1$H-NMR spectrum of compound **10a.**

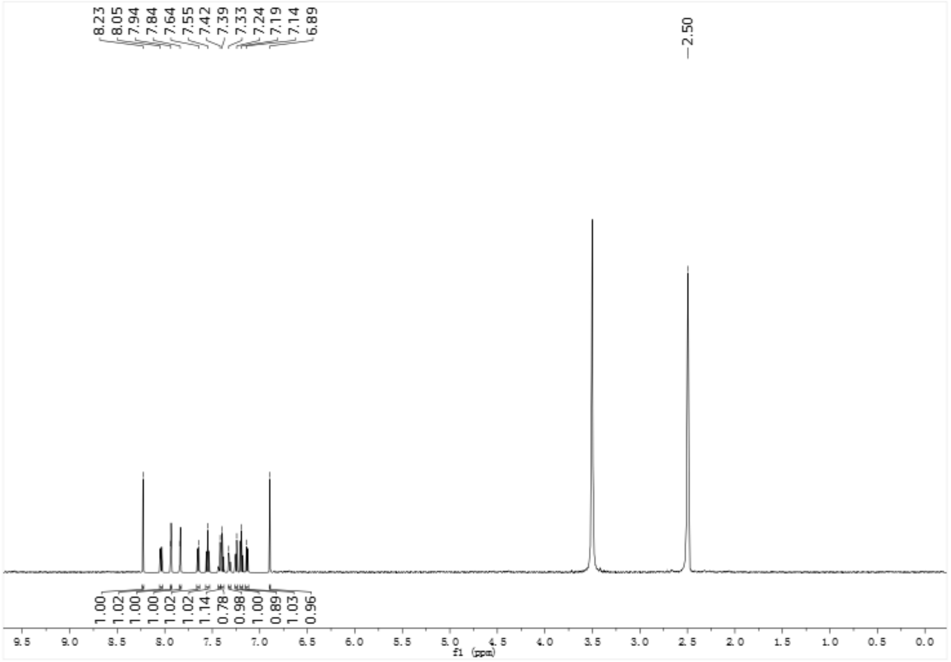


**Figure S20.** $^1$H-NMR spectrum of compound **10b.**

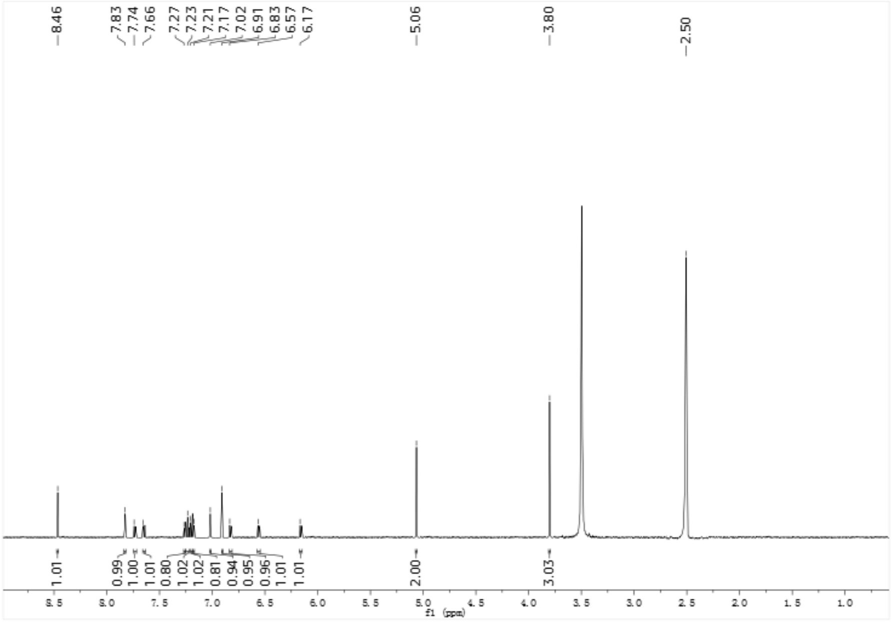


**Figure S21.** $^{1}$H-NMR spectrum of compound **11a.**

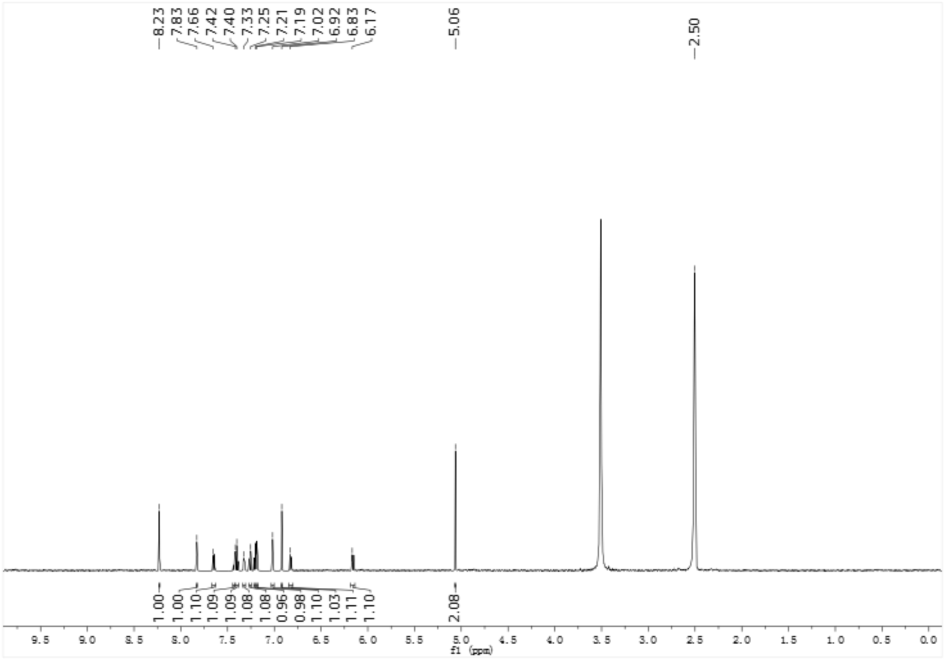


**Figure S22.** $^{1}$H-NMR spectrum of compound **11b.**

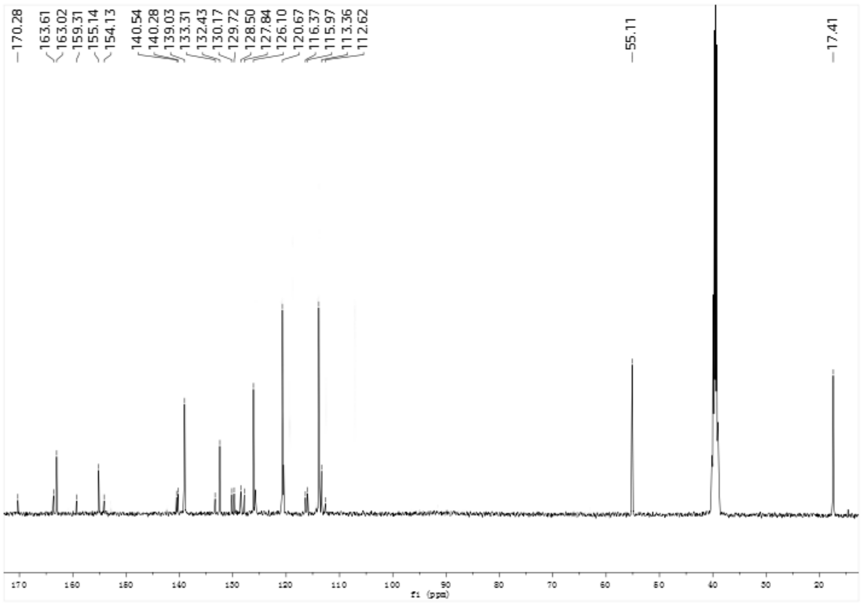


**Figure S23.** $^{13}$C-NMR spectrum of compound **H7.**

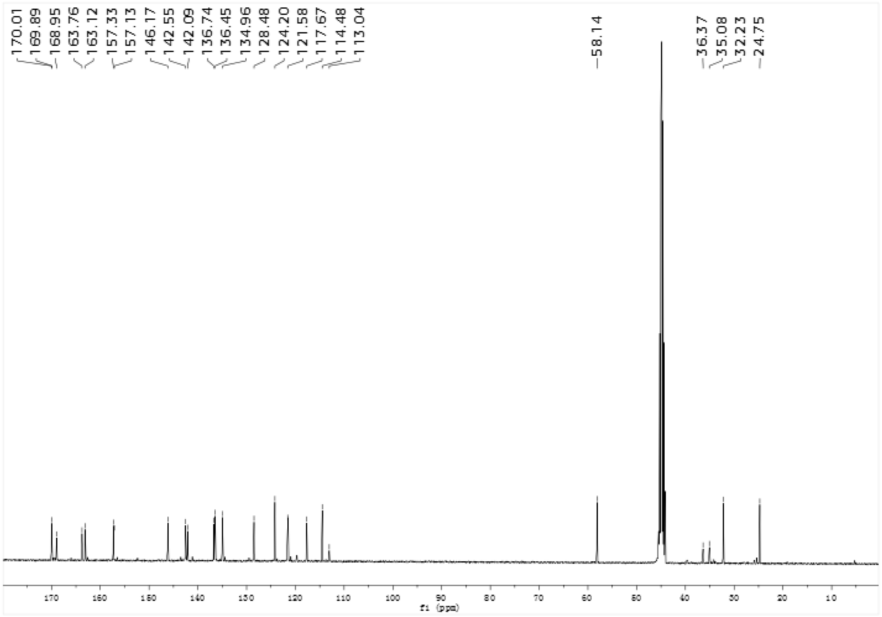


**Figure S24.** $^{13}$C-NMR spectrum of compound **H10.**

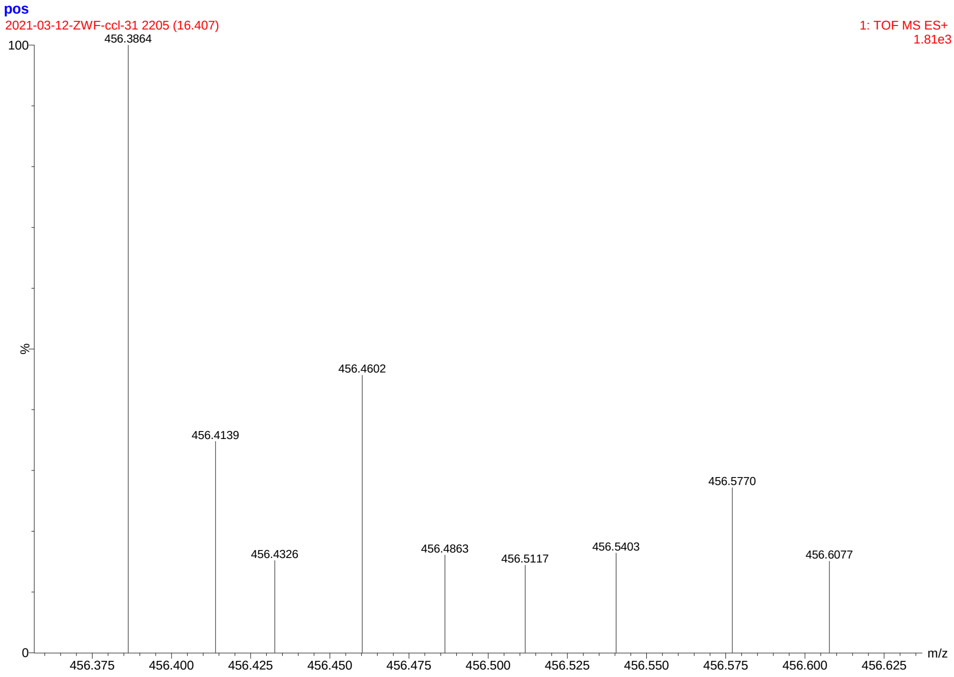


**Figure S25.** TOF MS of compound **H1.**

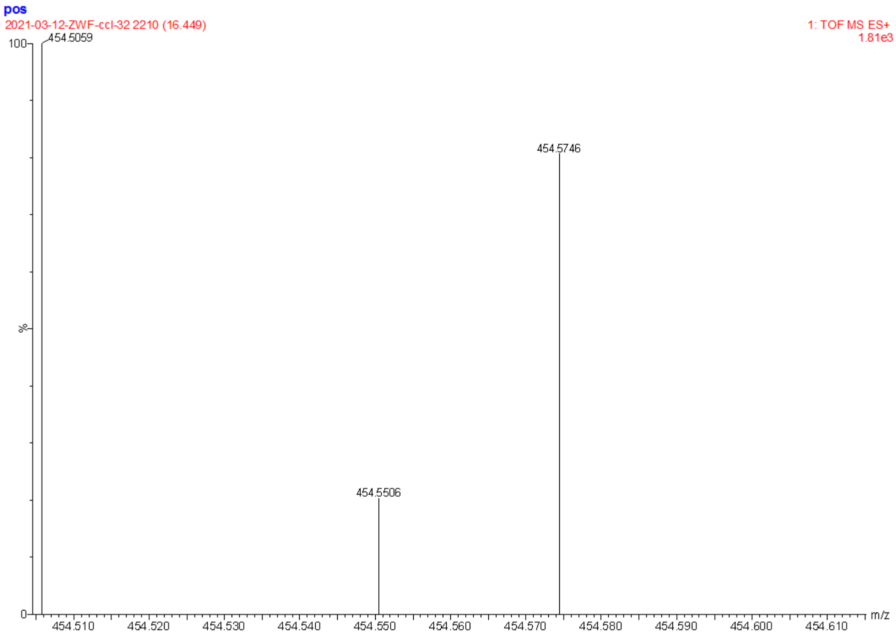


**Figure S26.** TOF MS of compound **H2.**

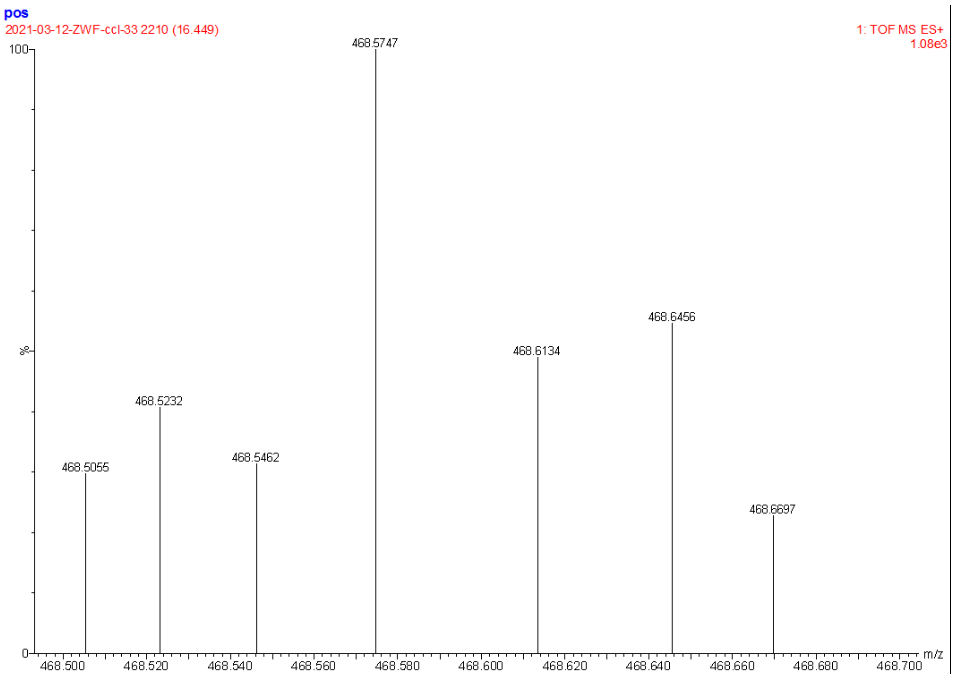


**Figure S27.** TOF MS of compound **H3.**

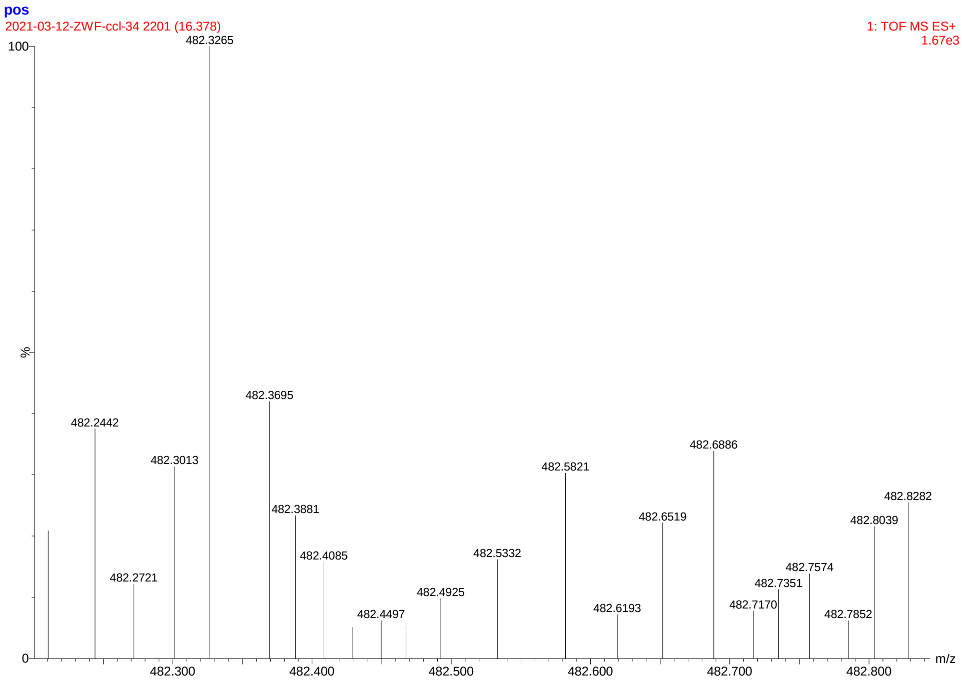


**Figure S28.** TOF MS of compound **H4.**

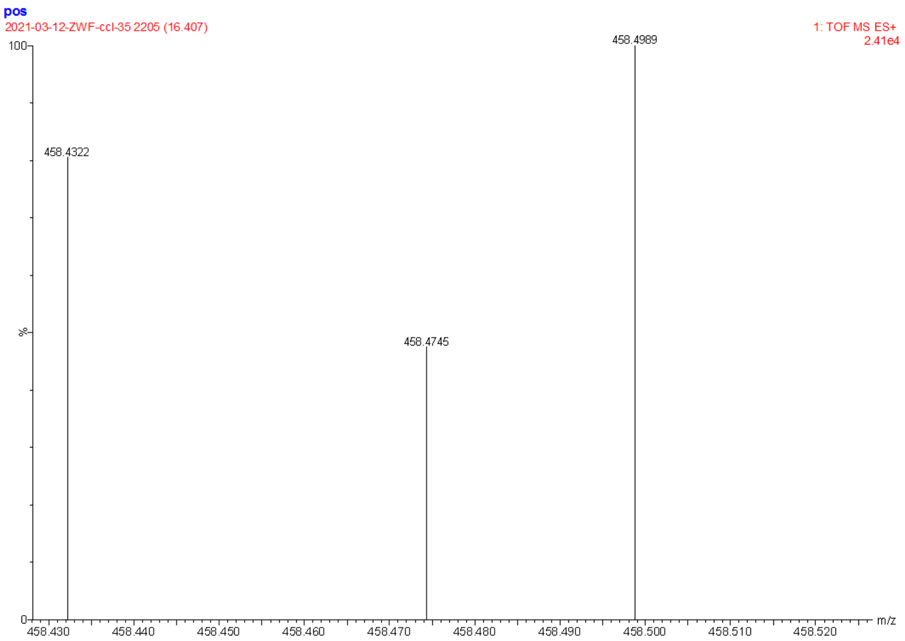


**Figure S29.** TOF MS of compound **H5.**

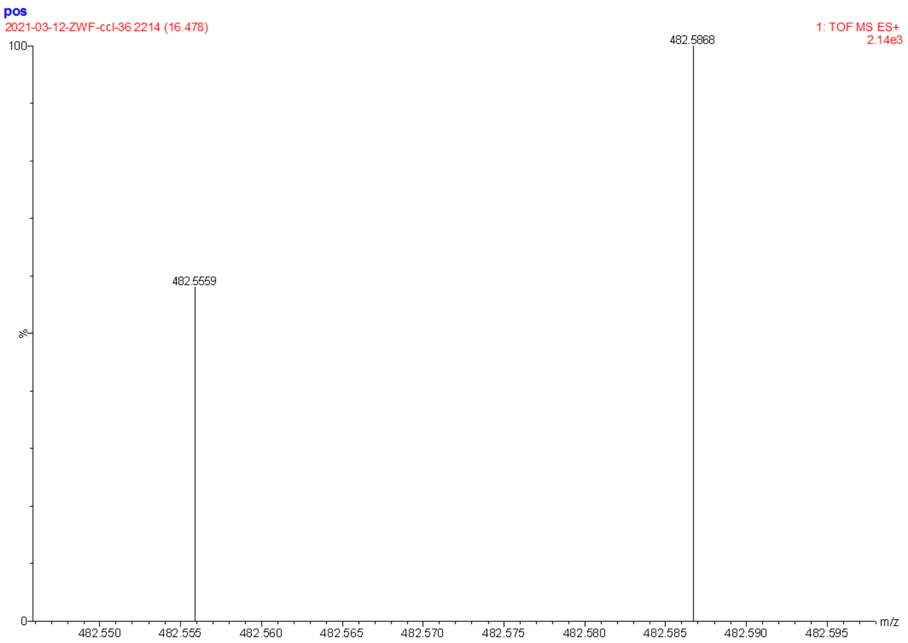


**Figure S30.** TOF MS of compound **H6.**

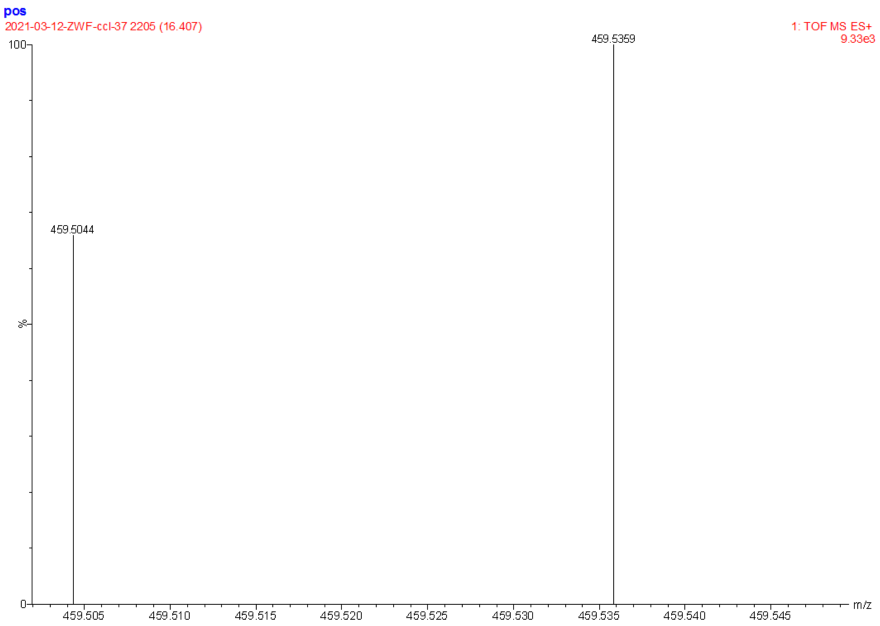


**Figure S31.** TOF MS of compound **H7.**

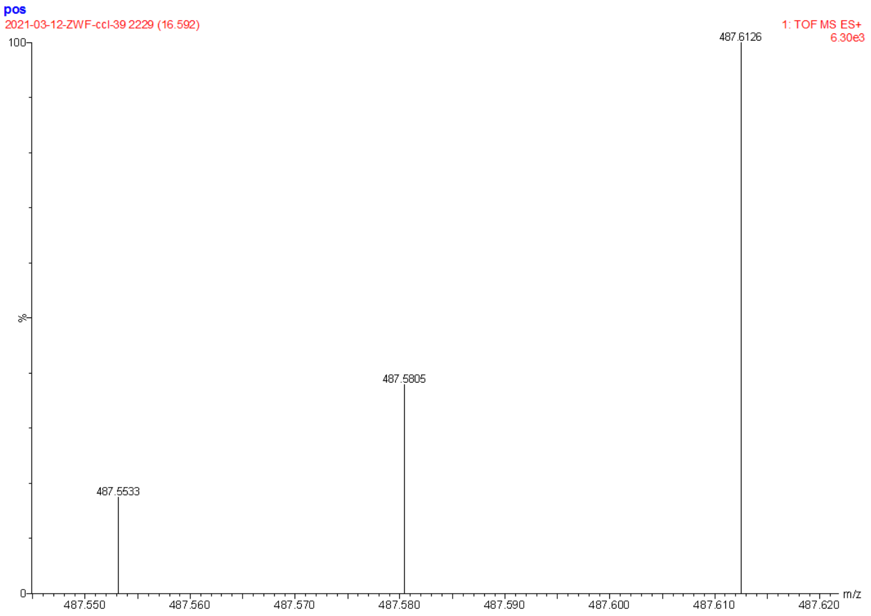


**Figure S32.** TOF MS of compound **H8.**

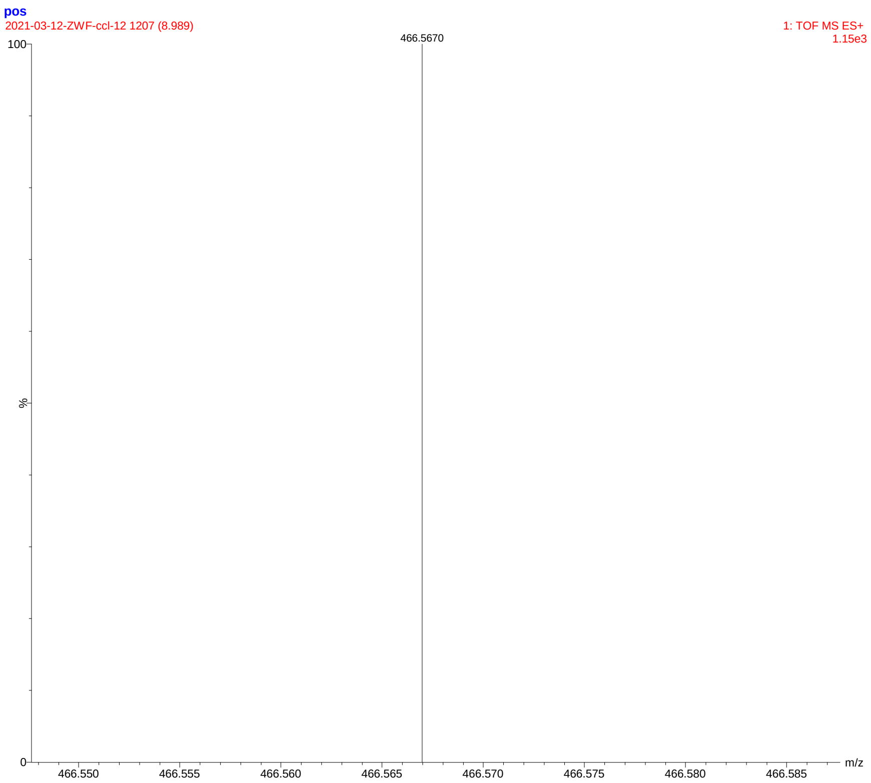


**Figure S33.** TOF MS of compound **H9.**

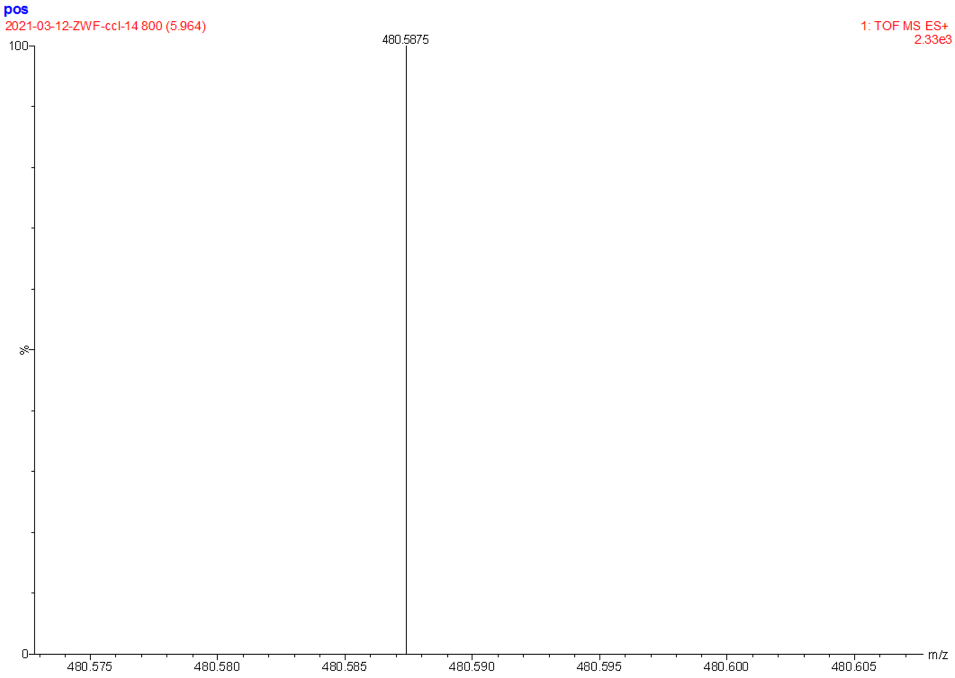


**Figure S34.** TOF MS of compound **H10.**

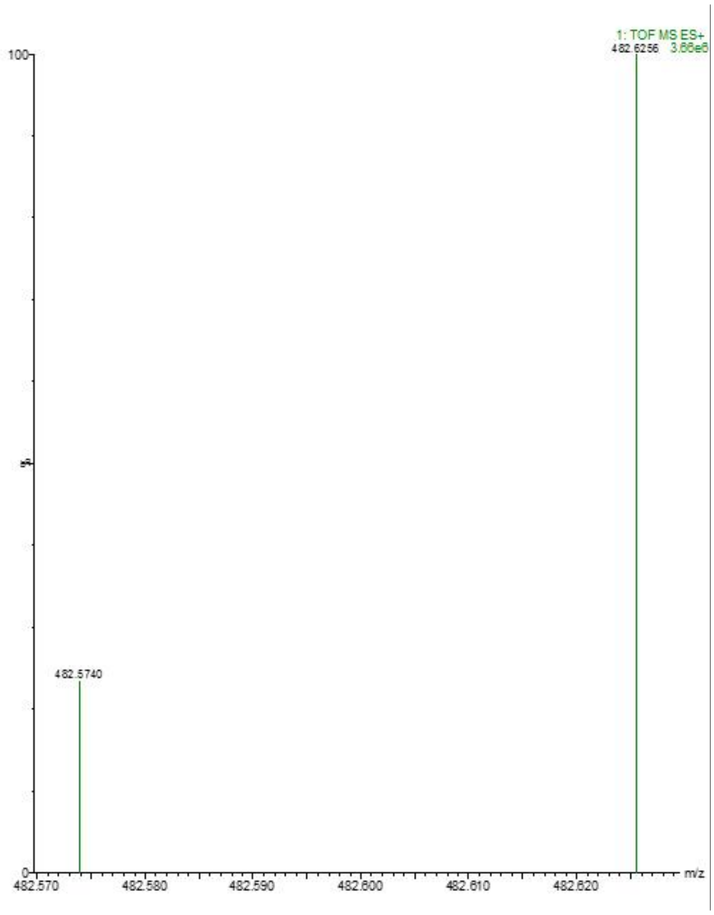


**Figure S35.** TOF MS of compound **H11.**

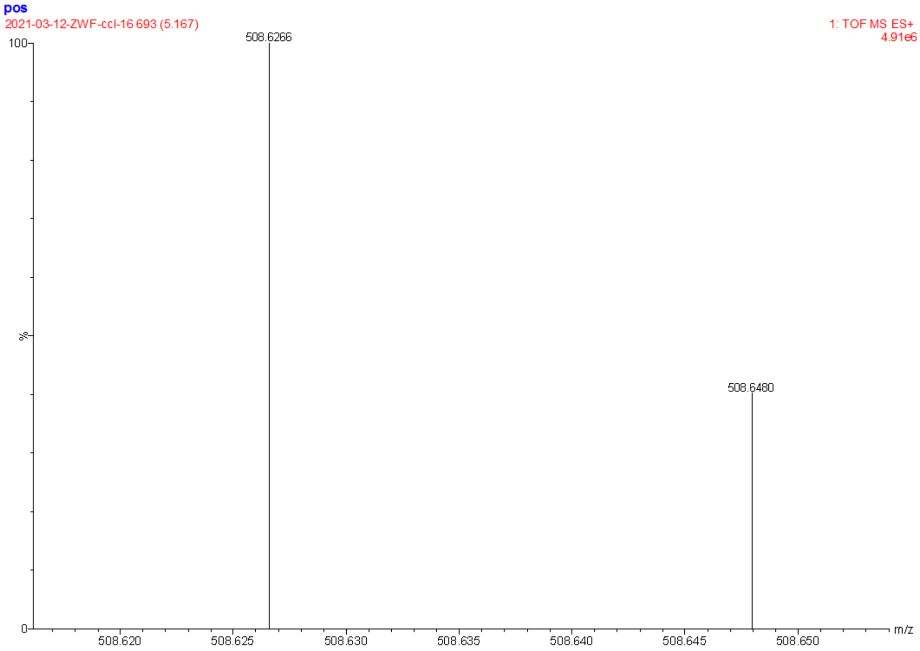


**Figure S36.** TOF MS of compound **H12.**

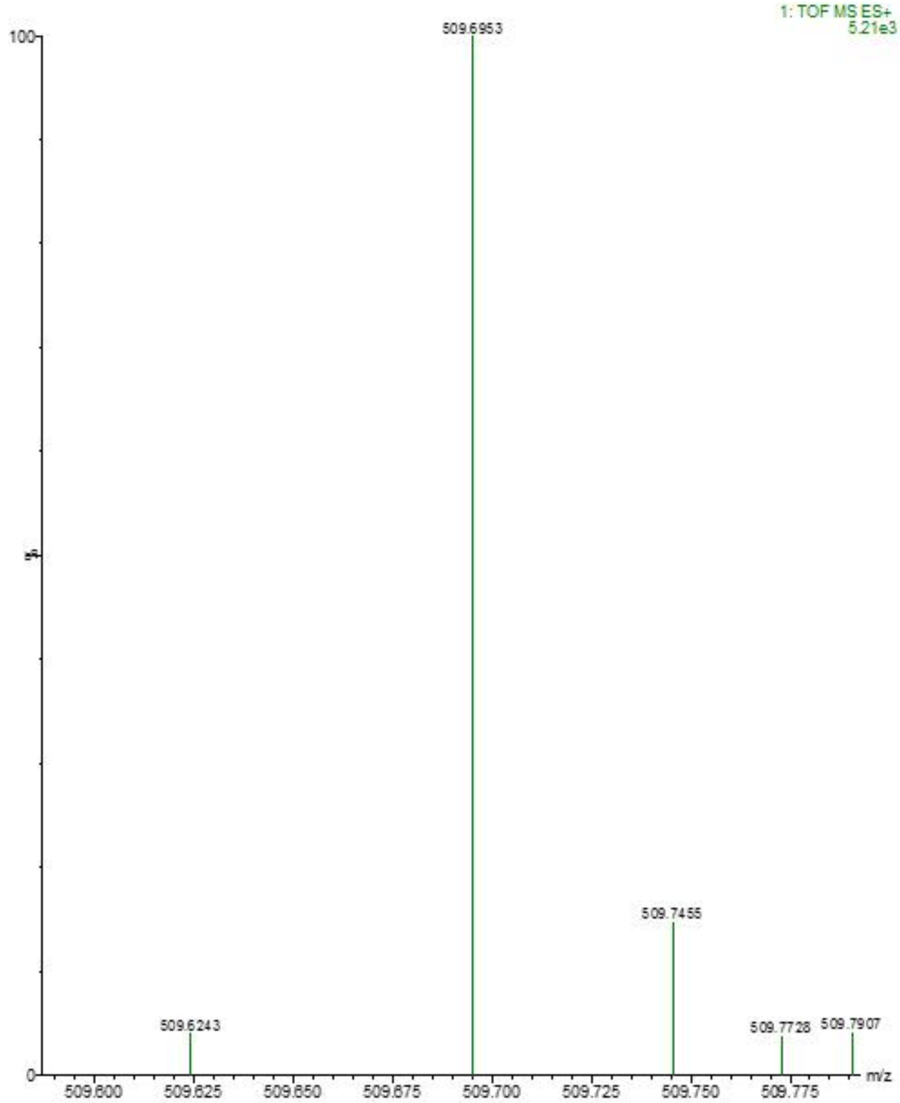


**Figure S37.** TOF MS of compound **H13.**

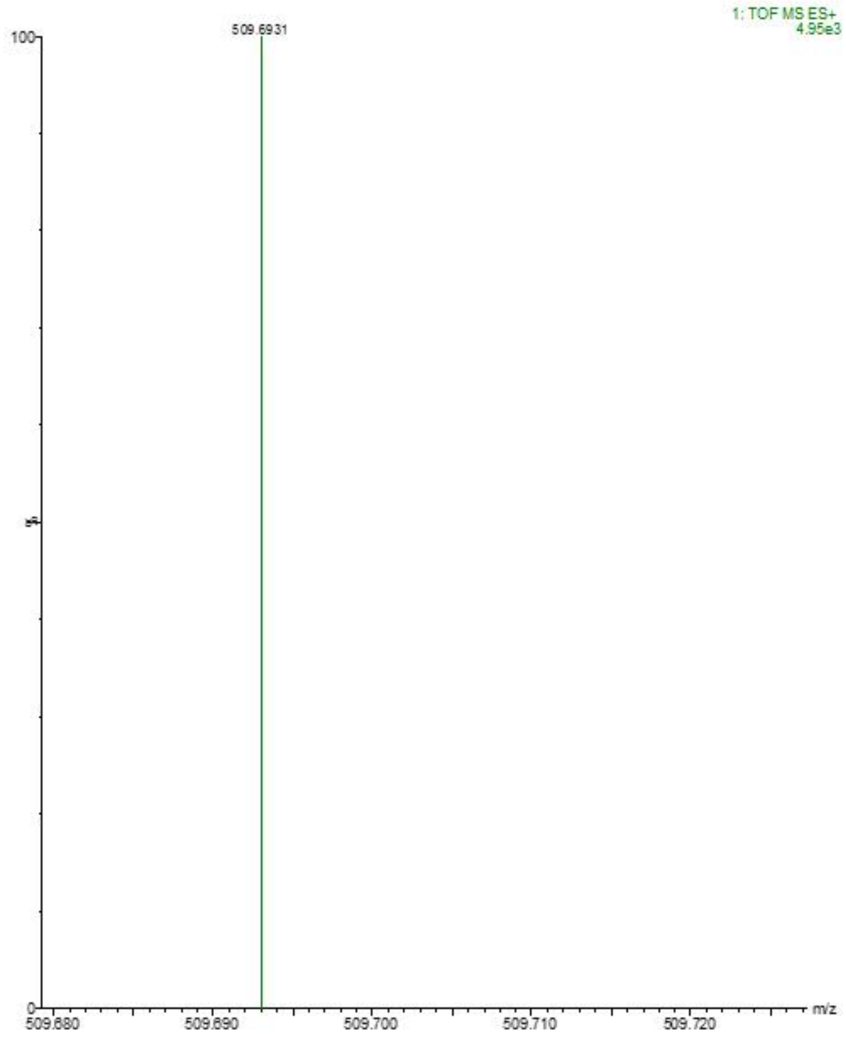


**Figure S38.** TOF MS of compound **H14.**

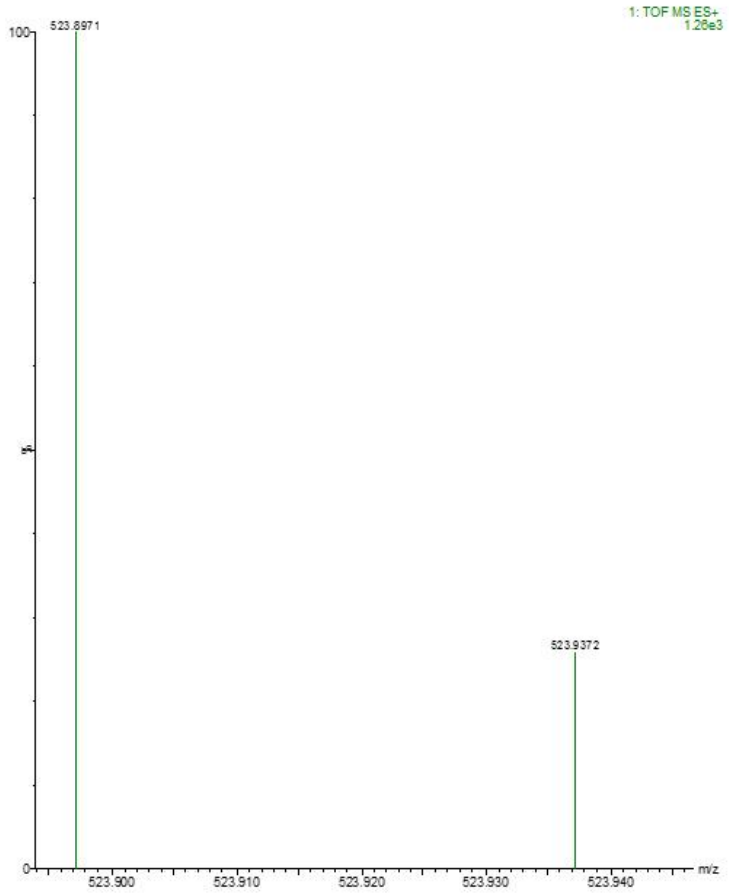


**Figure S39.** TOF MS of compound **H15.**

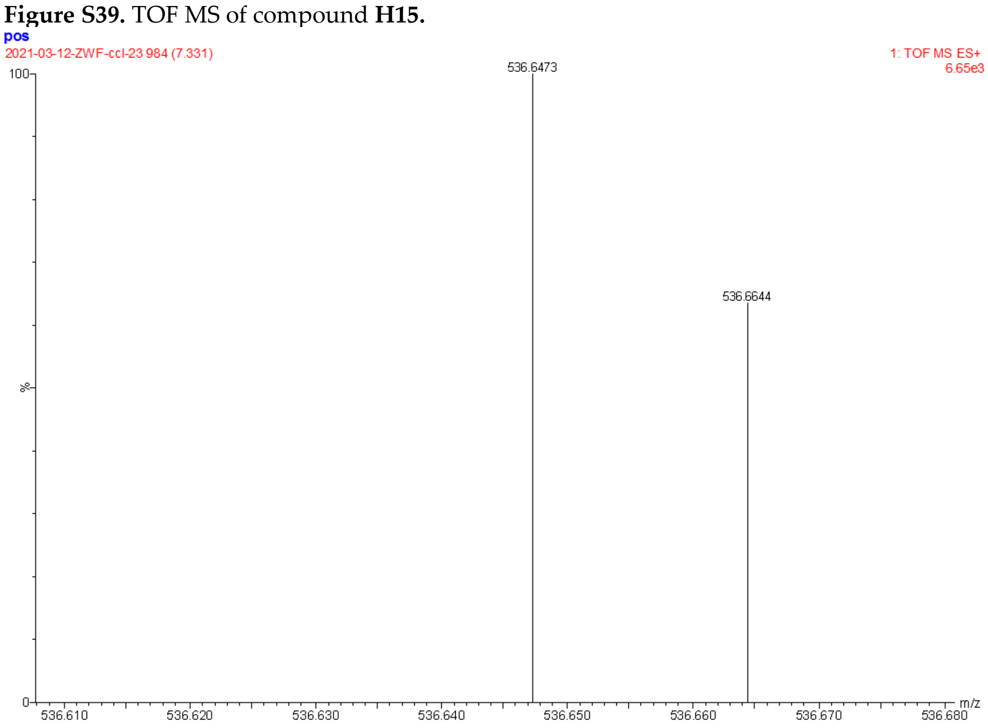


**Figure S40.** TOF MS of compound **H16.**

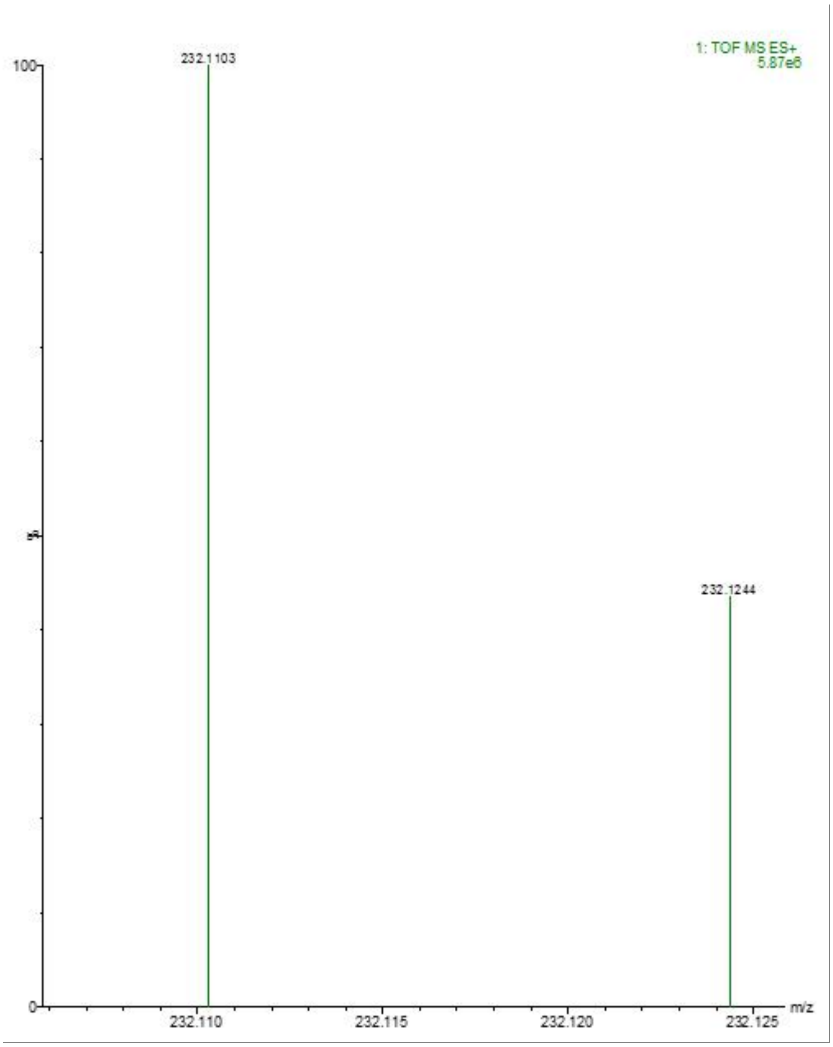


**Figure S41.** TOF MS of compound **8.**

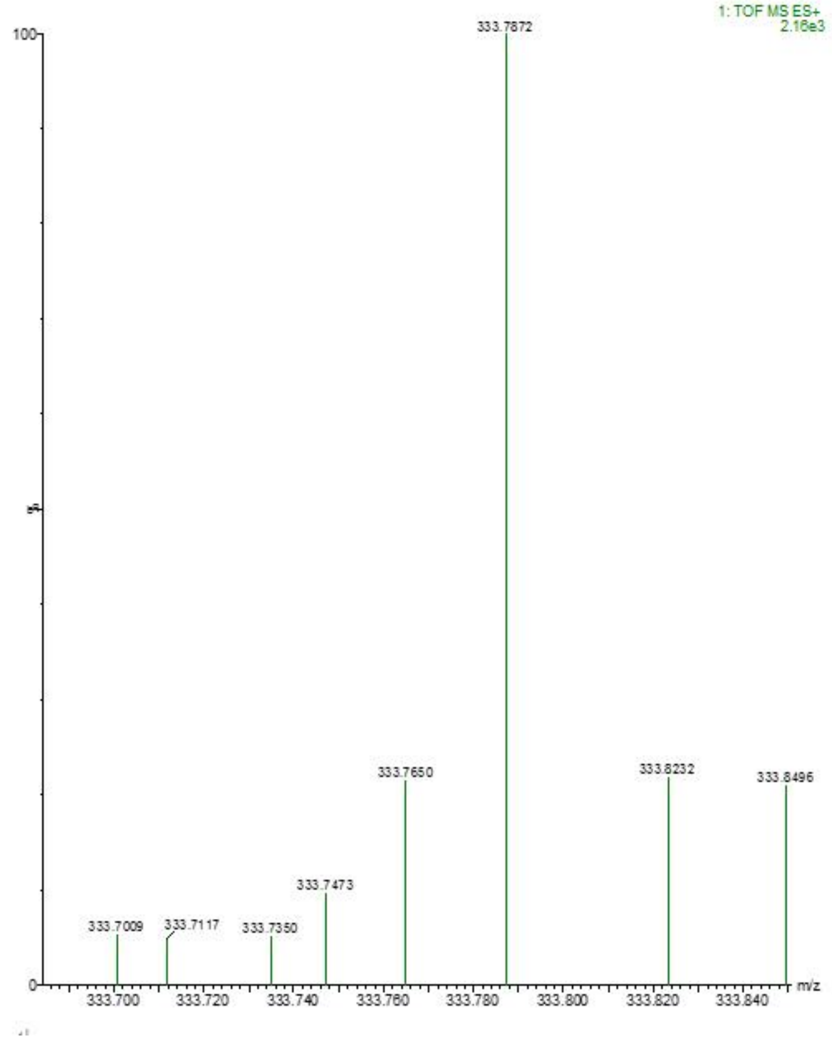


**Figure S42.** TOF MS of compound **9.**

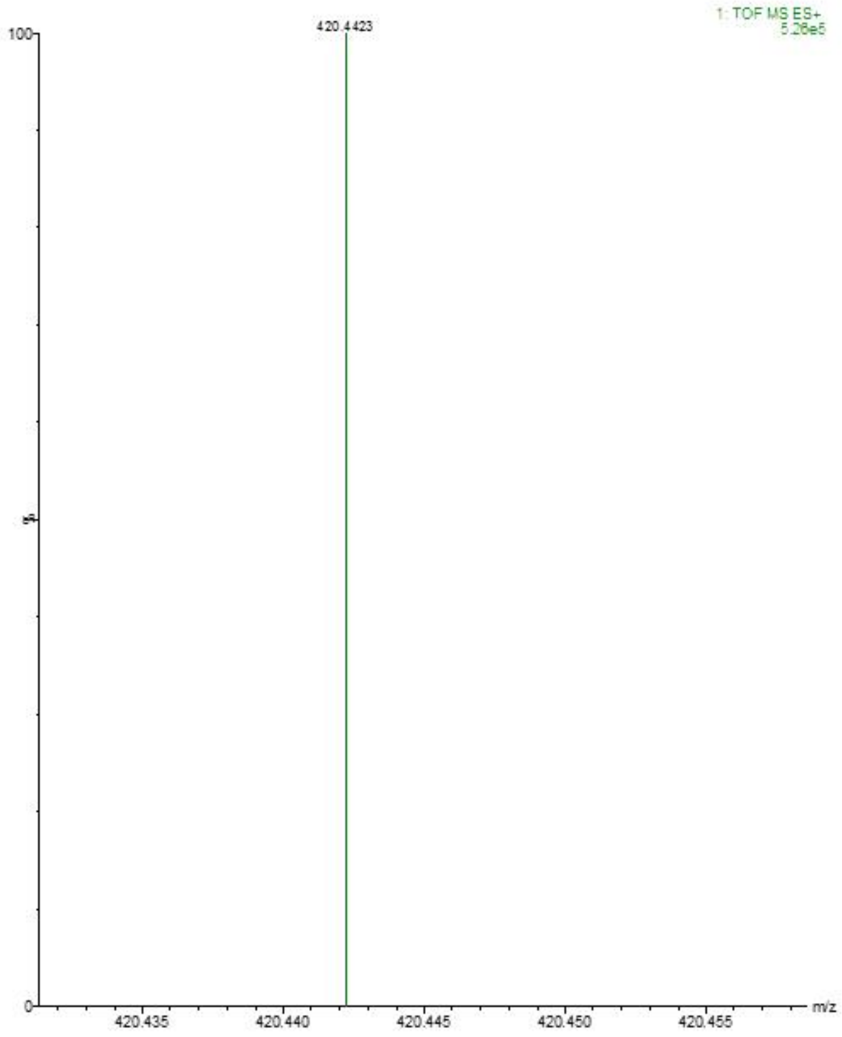


**Figure S43.** TOF MS of compound **10a.**

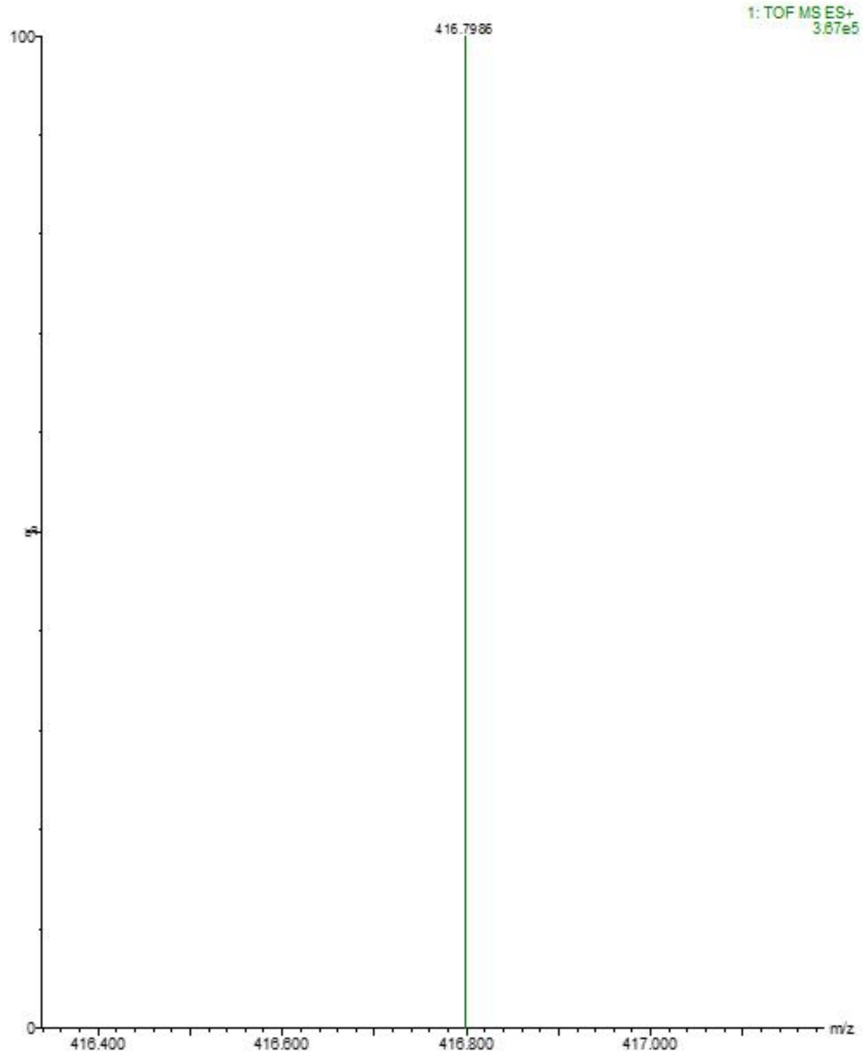


**Figure S44.** TOF MS of compound **10b.**

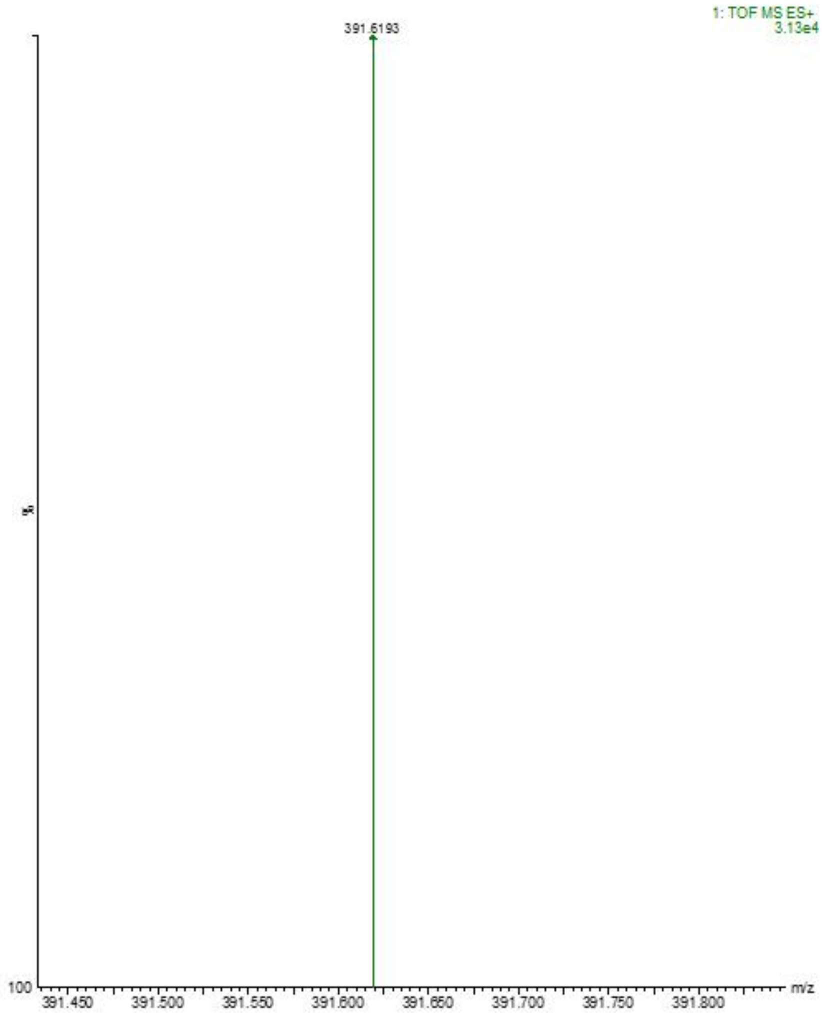


**Figure S45.** TOF MS of compound **11a.**

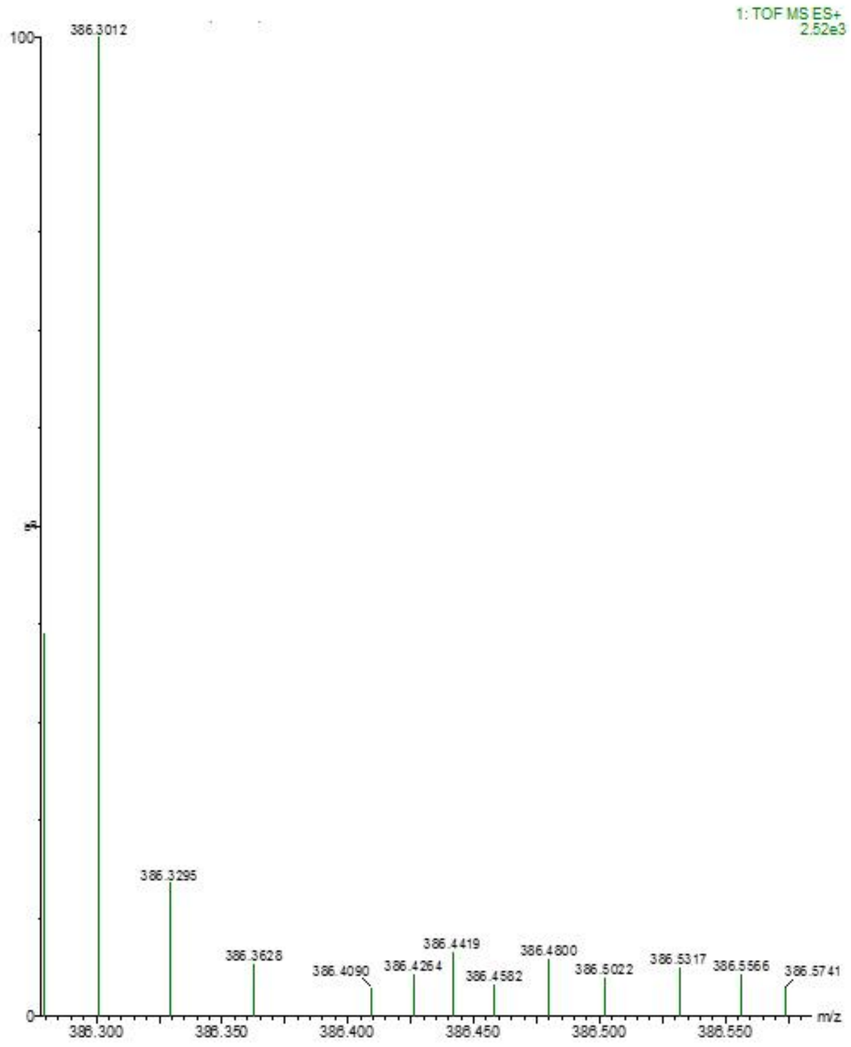


**Figure S46.** TOF MS of compound **11b.**

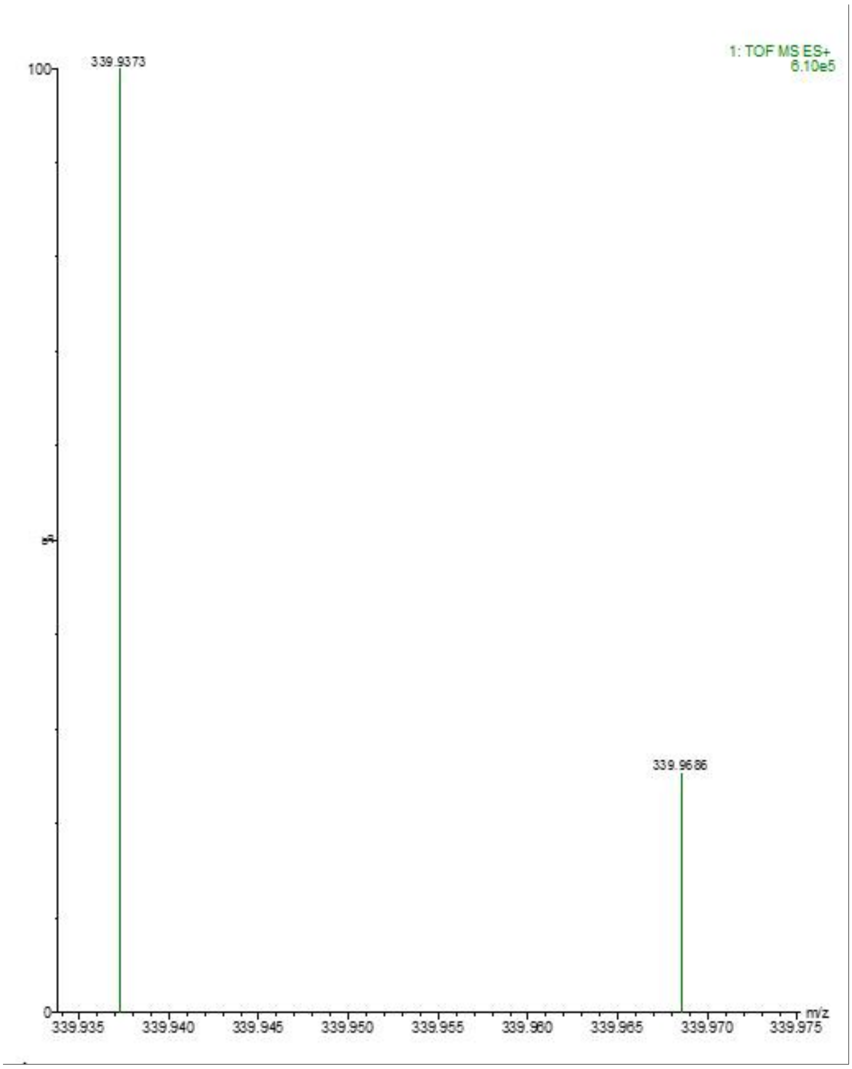


**Figure S47.** TOF MS of compound **17a.**

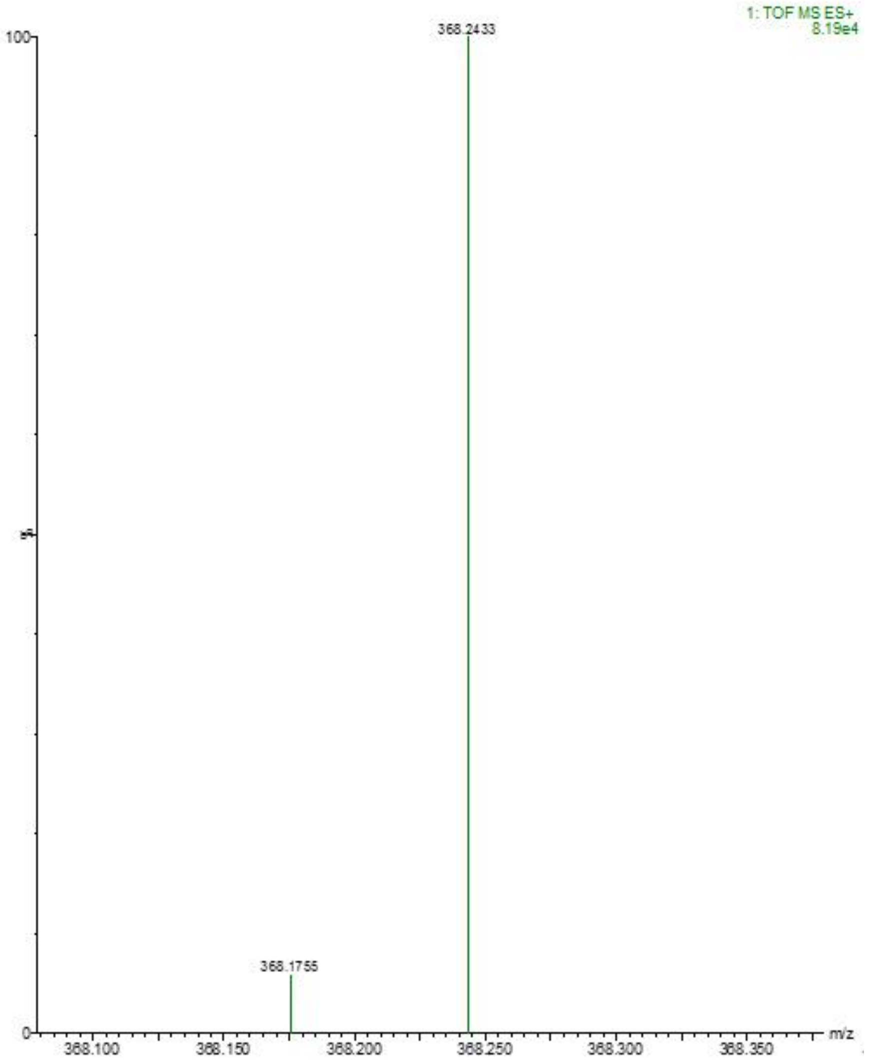


**Figure S48.** TOF MS of compound **17b.**

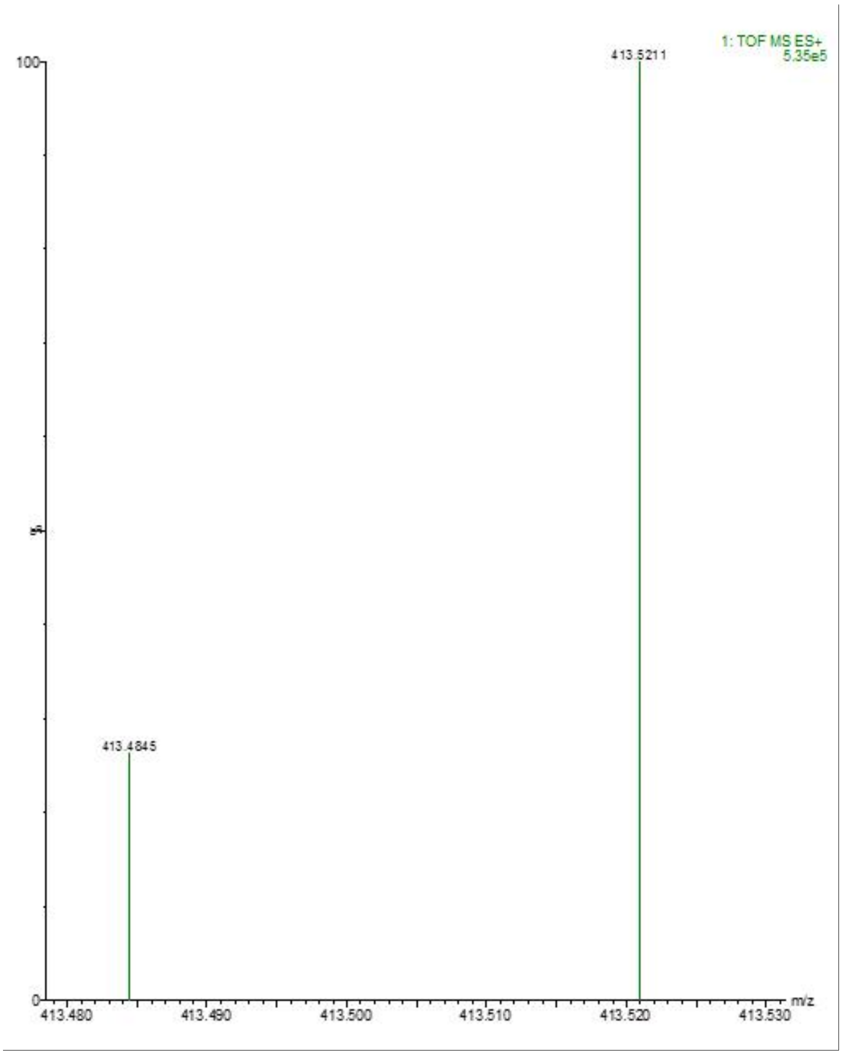


**Figure S49.** TOF MS of compound **18a.**

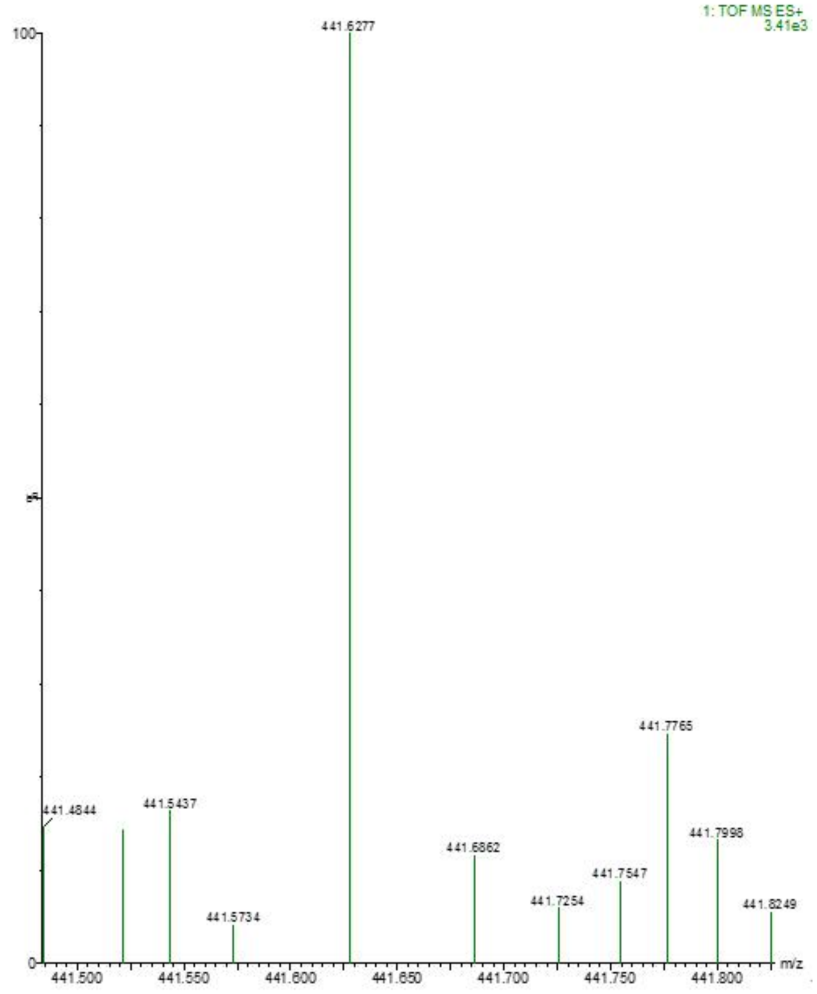


**Figure S50.** TOF MS of compound **18b.**
